# Origin, Evolution and Stability of Overlapping Genes in Viruses: A Systematic Review

**DOI:** 10.3390/genes12060809

**Published:** 2021-05-26

**Authors:** Angelo Pavesi

**Affiliations:** Department of Chemistry, Life Sciences and Environmental Sustainability, University of Parma, Parco Area delle Scienze 23/A, I-43124 Parma, Italy; angelo.pavesi@unipr.it; Tel.: +39-0521905647

**Keywords:** asymmetric evolution, codon usage, *de novo* protein creation, modular evolution, multivariate statistics, negative selection: phylogenetic distribution, positive selection, prediction methods, sequence-composition features, symmetric evolution, virus evolution

## Abstract

During their long evolutionary history viruses generated many proteins *de novo* by a mechanism called “overprinting”. Overprinting is a process in which critical nucleotide substitutions in a pre-existing gene can induce the expression of a novel protein by translation of an alternative open reading frame (ORF). Overlapping genes represent an intriguing example of adaptive conflict, because they simultaneously encode two proteins whose freedom to change is constrained by each other. However, overlapping genes are also a source of genetic novelties, as the constraints under which alternative ORFs evolve can give rise to proteins with unusual sequence properties, most importantly the potential for novel functions. Starting with the discovery of overlapping genes in phages infecting *Escherichia coli*, this review covers a range of studies dealing with detection of overlapping genes in small eukaryotic viruses (genomic length below 30 kb) and recognition of their critical role in the evolution of pathogenicity. Origin of overlapping genes, what factors favor their birth and retention, and how they manage their inherent adaptive conflict are extensively reviewed. Special attention is paid to the assembly of overlapping genes into *ad hoc* databases, suitable for future studies, and to the development of statistical methods for exploring viral genome sequences in search of undiscovered overlaps.

## 1. Introduction

Modification of existing genes, such as duplication followed by functional divergence, fusion (two adjacent genes fuse into a single gene), fission (a single gene splits into two genes), exon shuffling (rearrangement of protein modules), or horizontal gene transfer (gene exchange between unrelated species), is a common mechanism by which new genes arose during the evolution of living organisms [1,2,3,4]. However, genes can also originate *de novo* by taking place in non-coding regions, such as intergenic regions or introns [5,6].

During their long evolutionary history viruses generated many proteins *de novo* by a mechanism called “overprinting” [7]. Overprinting is a process in which critical nucleotide substitutions in a pre-existing gene can induce the expression of a novel protein by translation of an alternative open reading frame (ORF), while preserving the function of pre-existing gene [8]. It is thought that most overlapping genes evolve by this mechanism, and that consequently each overlap contains one ancestral frame and one originated *de novo* [9]. It is also believed that overprinting is a source of genetic novelties, because the *de novo* proteins, unlike the ancestral ones, usually lack any remote homologs in databases [10].

**Figure 1:**
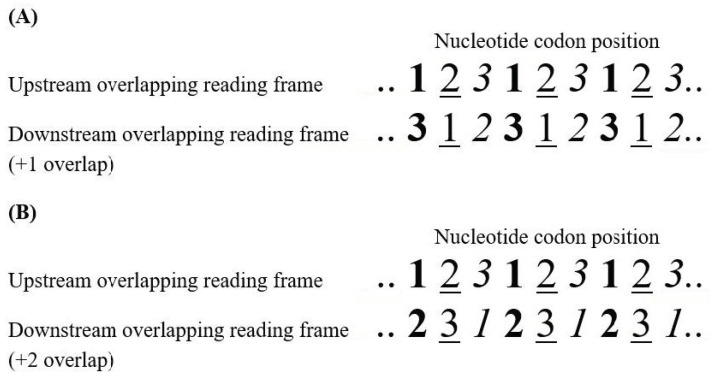
Orientation of same-strand overlapping genes. (**A**) Overlapping gene with the downstream frame shifted one nucleotide 3′ with respect to the upstream frame. There are 3 types of codon position (cp): cp13 (bold character), in which the first codon position of the upstream frame overlaps the third codon position of the downstream frame; cp21 (underlined character), in which the second codon position of the upstream frame overlaps the first codon position of the downstream frame; cp32 (italic character), in which the third codon position of the upstream frame overlaps the second codon position of the downstream frame. (**B**) Overlapping gene with the downstream frame shifted two nucleotides 3′ with respect to the upstream frame. There are 3 types of codon position (cp): cp12 (bold character), in which the first codon position of the upstream frame overlaps the second codon position of the downstream frame; cp23 (underlined character), in which the second codon position of the upstream frame overlaps the third codon position of the downstream frame; cp31 (italic character), in which the third codon position of the upstream frame overlaps the first codon position of the downstream frame. According to the genetic code, a nucleotide substitution at first codon position causes an amino acid change in 95.4% of cases, at second position in 100% of cases, and at third position in 28.4% of cases.

Most of new genes originated by overprinting are expressed by the sense strand. They are classified as same-strand, or parallel, overlapping genes because of transcription from the same strand of DNA. They are usually denoted as +1 overlapping genes, when the *de novo* frame is shifted one nucleotide 3′ with respect to the ancestral frame (Figure 1A), or as +2 overlapping genes when the *de novo* frame is shifted two nucleotides 3′ (Figure 1B). According to genetic code, 71.6% of substitutions in the third codon position are synonymous, compared to only 0 and 4.6% of substitutions in the second and first codon positions respectively. In overlapping genes, therefore, a nucleotide substitution that is synonymous in one frame is highly likely to be non-synonymous in the alternative frame.

One of the reasons why overlapping genes have long attracted attention of researchers is that they represent an intriguing example of adaptive conflict. Indeed, they simultaneously encode two proteins whose freedom to change is constrained by each other, which would be expected to severely reduce the ability of the virus to adapt. On the other hand, the unusual constraints under which alternative ORFs evolve can give rise to proteins with unusual sequence properties, most importantly the potential for novel structural folds and mechanisms of action.

This review deals with the origin of overlapping genes, what factors favor their birth and retention, how they influence the evolution of viral genome, and how they manage their inherent adaptive conflict. The review is focused on overlapping genes from small viruses (genomic length below 30 kb), in which both members of the pair are known to be expressed during infection. Special attention is paid to the genealogy of the overlap, that is inferring which frame is ancestral and which one is de novo. Special attention is paid to the assembly of overlapping genes into *ad hoc* databases, suitable for future studies, and to the development of statistical methods for exploring viral genome sequences in search of undiscovered overlapping coding regions.

## 2. Discovery of Overlapping Genes and Evolutionary Implications

Overlapping genes, also called “dual-coding genes”, were first discovered by Barrell et al. [11] in the genome of ΦX174, a small single-stranded DNA virus (5386 nt) that infects *Escherichia coli*. Analysis of the fully sequenced genome revealed that it contains, thanks to overprinting, 15% more coding ability than a co-linear relationship between nucleotide and protein sequences would suggest [12].

Genome sequence analysis of ΦX174 showed that there are two types of overlaps: in “internal overlaps” one overlapping gene is contained entirely within the other gene (e.g., gene E is nested within gene D) whereas “terminal overlaps” involve only the 3′ terminal region of one gene and the 5′ start region of another (e.g., the 3′ end of gene A overlaps the 5′ end of gene K) [12]. The strength of selection pressure acting on the gene overlap was estimated by a mathematical model, which pointed out a strong reduction of amino acid changes (at most 40 or 50%) in the overlapping genes B and D of ΦX174 [13].

By sequence analysis of the paired overlapping genes D and E, Fiddes and Godson proposed a simple method to predict the genealogy of the overlap [14]. Genealogy means to recognize which frame is ancestral and which frame is *de novo*. The authors first found that the genome of ΦX174 is rich in T nucleotides (31%) and these tend to occur at third codon position. They then found that in the region of D overlapping E (279 nt) the high incidence of T-ending codons is a feature of the frame D (39%) rather than the frame E (14%). Based on this finding, D was predicted as the ancestral gene and E as the *de novo* gene.

In addition, displacement of the high T content from the third codon position in frame D to the second codon position in frame E yields a high incidence of codons that specify leucine, one of the most hydrophobic amino acids, in frame E. The high content of leucine in protein E is mainly localized within a transmembrane domain, which induces lysis of the cell host *Escherichia coli* [15] by inhibiting biosynthesis of cell wall [16]. This finding suggests that de novo protein creation can be a significant factor in the evolution of pathogenicity.

The Fiddes’s method to predict the genealogy of the overlap [14] was improved by means of a correlation analysis of the codon usage [17]. It was based on the assumption that the ancestral gene, which has co-evolved with the other viral genes over a long period of time, has a distribution of synonymous codons closer to that of the viral genome than the *de novo* gene. The codon-usage correlation analysis of ΦX174 demonstrated that E and K are *de novo* overlapping proteins and that the C-terminal region of protein A is a *de novo* overlapping extension [17].

**Figure 2:**
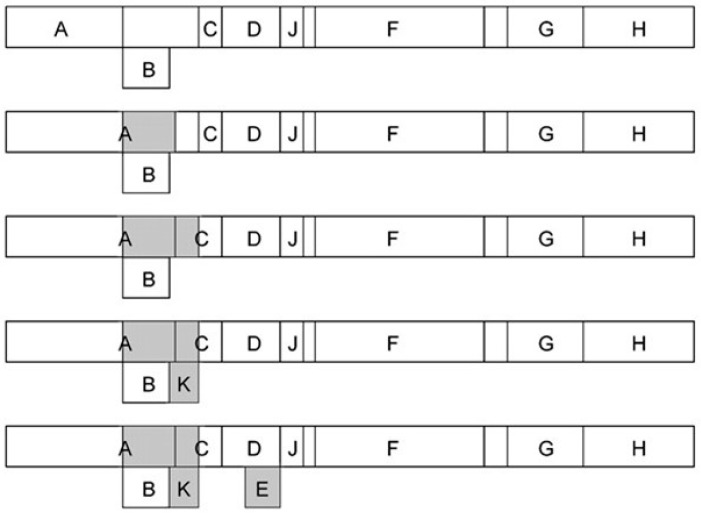
Increase in the genome information content during the evolution of microviruses (family *Microviridae*). The nomenclature of genes, from A to J, is that originally proposed in ΦX174 [11,12,13]. Empty boxes indicate ancestral pre-existing genes, while grey boxes indicate the new genes (or gene regions) that originated by overprinting. Figure reproduced from [18] with the permission of the Microbiology Society.

When applied to ΦX174, α3 and G4 (the three evolutionary clades of the genus *Microvirus*, family *Microviridae*), the codon-usage correlation analysis predicted a gradual increase in the genome information content due to overprinting [18]. It predicted an ancestral genome having only single-coding genes, whose coding capacity increased over time due to the birth of novel overlapping coding regions (Figure 2). This fine evolutionary process led to the present genome, which contains two *de novo* overlapping genes (K and E) and two *de novo* overlapping extensions of genes A and C.

As said in introduction, an intriguing paradox of overlapping genes is that the biological information in the encoded proteins is strongly interdependent, yet each of the two proteins has evolved to its own well-defined function. Sander and Schultz [19] developed a mathematical model and applied it to the overlapping proteins A and B of ΦX174. The model postulated that the paradox can be explained by assuming sufficiently large degeneracy of the information content of amino acid sequences with respect to function.

## 3. *De Novo* Overlapping Genes Show a Restricted Phylogenetic Distribution and Encode Accessory Proteins

In 1992, Keese and Gibbs published a seminal paper [9] in which the birth of new genes by overprinting was described as a continuous, and significant, evolutionary process. They proposed a new method to predict the genealogy of overlapping genes. It is based on the assumption that the protein with the most restricted phylogenetic distribution is encoded by the *de novo* frame, while that with the widest distribution is encoded by the ancestral frame.

**Figure 3:**
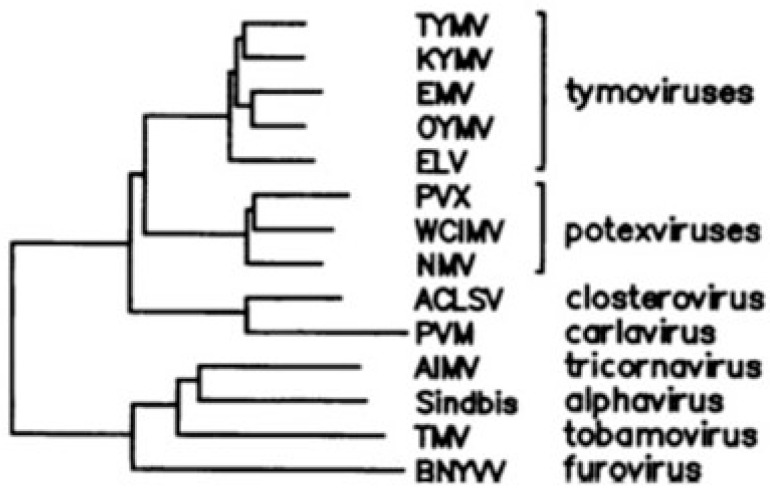
Dendrogram of the methyltransferase-like domain of replicase from Turnip yellow mosaic virus (TYMV), Kennedya yellow mosaic tymovirus (KYMV), Eggplant mosaic tymovirus (EMV), Ononis yellow mosaic tymovirus (OYMV), Erysimum latent tymovirus (ELV), Potato X potexvirus (PVX), White clover mosaic potexvirus (WClMV), Narcissus mosaic potexvirus (NMV), Apple chlorotic leaf spot closterovirus (ACLSV), Potato M carlavirus (PVM), Alfalfa mosaic alfamovirus (AIMV), Tobacco mosaic tobamovirus (TMV), and Beet necrotic yellow vein furovirus (BNYVV). The overlapping ORF encoding a movement protein (entirely nested within replicase) is a genetic novelty unique to tymoviruses. Figure reproduced from [9] with the permission of the authors.

As an example to explain the phylogenetic method, the genome of tymoviruses contains a large dual-coding region in which the 5′ one-third of replicase, encoding a methyltransferase domain, overlaps an ORF that encodes a movement protein necessary for viral spread [20]. While the methyltransferase domain has a wide phylogenetic distribution, including the closely related sister groups of potexviruses and closteroviruses or outgroups such as tricornaviruses and furoviruses, the movement protein is unique to tymoviruses (Figure 3).

Based on this finding, Keese and Gibbs inferred that replicase is the ancestral gene and that the overlapping ORF arose later, de novo, after the evolutionary divergence between tymoviruses and potexviruses. It is unlikely, indeed, that this ORF was present earlier but was subsequently lost in all virus groups with the exception of tymoviruses. It follows that the genome region of potexviruses homologous to the gene overlap unique to tymoviruses should have sequence-composition features typical of a “pre-overlapping” coding region.

Using the phylogenetic method, Rancurel et al. [21] were able to recognize the ancestral and the *de novo* frame for 17 pairs of overlapping genes, covering a wide evolutionary range of RNA viruses. Almost all de novo frames resulted to encode accessory proteins, rather than proteins central to viral replication or to the structure of capsid. “Accessory” does not mean that they are dispensable in vivo, because most novel proteins play an important role in viral pathogenicity or spread. Indeed, six de novo proteins promote a systemic diffusion of infection in plants [20,22,23,24,25], for example by binding viral RNA and forming protective ribonucleoprotein complexes [26]. Two de novo proteins contribute to evade or counteract the innate host defense, acting as inhibitor of interferon response [27] or suppressor of RNA silencing [28].

**Figure 4:**
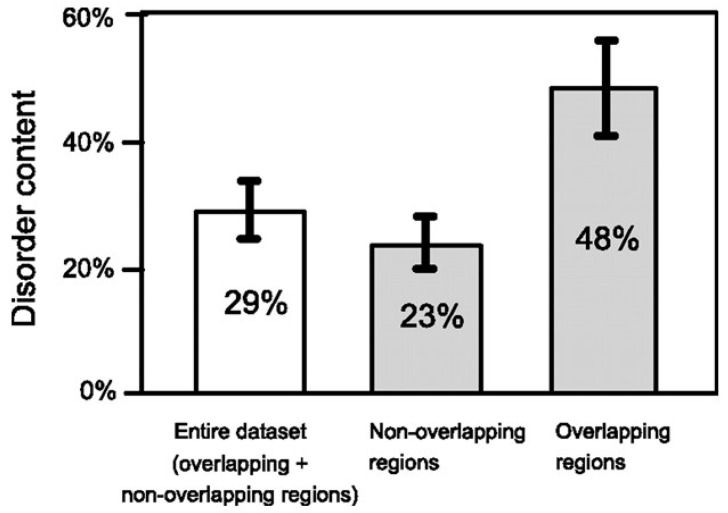
Predicted disorder content of proteins encoded by overlapping genes. The error bars correspond to a 95% confidence interval. Figure reproduced from [21] with the permission of the American Society of Microbiology.

The same study [21] demonstrated that most de novo proteins have a sequence composition globally biased toward disorder-promoting amino acids and that overlapping proteins are predicted to contain significantly more structural disorder than non-overlapping proteins (the term disorder applies to proteins which lack a stable secondary and tertiary structure, at least in the absence of a binding partner) (Figure 4). Based on the notion that disordered proteins are generally subjected to less structural constraint than ordered ones [29], Rancurel et al. proposed that presence of disorder in one or both overlapping proteins could relieve the evolutionary constraints imposed by the overlap.

This feature was further investigated by Willis and Masel [30], who analyzed a dataset of 92 overlapping genes spanning 33 viral families, 47 of them with a predicted ancestral and *de novo* frame. In accordance to [21], the authors found that the mean predicted value of the intrinsic structural disorder (ISD) in overlapping proteins is significantly higher than that in non-overlapping proteins. In addition, they found that the *de novo* proteins have a higher ISD than the ancestral ones, but this feature is specific to overlapping genes with a *de novo* frame shifted two nucleotides 3′ (+2 overlap) with respect to the ancestral frame.

The Willis study also demonstrated that the majority of overlapping genes (75%) shows a de novo frame shifted one nucleotide 3′ (+1 overlap) with respect to the ancestral frame. This feature was stronger for internal overlaps, in which one gene is completely contained within its overlapping partner, and was not found for terminal overlaps, in which the 3′ end of the upstream gene overlaps with the 5′ end of the downstream member of the pair. The prevalence of +1 gene births, despite the advantage of higher ISD in +2 gene births, was explained by the mutation bias. By sequence analysis of a control set of non-overlapping genes, Willis and Masel found that +1 frameshifts are evolutionary advantaged, because they yield significantly more ATG start codons (1 per 27 codons) than +2 frameshifts (1 per 111) and slightly fewer termination codons (1 per 14 codons) than +2 frameshifts (1 per 11).

## 4. Advanced Evolutionary Studies and Creation of a Curated Dataset of Overlapping Genes with Known Expression

As reported in the previous paragraph, identifying which frame of a gene overlap is ancestral and which one is de novo can be done by assessing their phylogenetic distribution (the frame phylogenetically most restricted is assumed to be the de novo one). This approach is simple and reliable but is not applicable to cases where the two frames have an identical phylogenetic distribution.

To overcome this drawback, Pavesi et al. [31] developed a new method to identify the de novo proteins. Like the previous ones [14,17,18], the method relied on the codon usage but was statistically more robust (the method assumes that the novel frame has a codon usage significantly less related to that of viral genome than the ancestral frame). It used as benchmark a reference dataset of 27 overlapping genes whose genealogy was predicted using the phylogenetic criterion. For each overlap, the method calculated: (i) the correlation coefficient (*r*_1_) between the codon usage of the ancestral frame and that of the viral genome; (ii) the correlation coefficient (*r*_2_) between the codon usage of the novel frame and that of the viral genome. Using the t-Hotelling test, the method evaluated the significance of the difference between *r*_1_ and *r*_2_, and predicted the genealogy of the overlap only in the case of *r*_2_ significantly lower (and not simply lower) than *r*_1_.

The method was applied to seven cases of overlap in which both frames have the same phylogenetic distribution, making the phylogenetic criterion not applicable. It demonstrated that the codon usage of overlapping frames was significantly different (or very close to significance) in only three cases: the overlap Tax protein/Rex protein of *Deltaretrovirus* and the overlap replicase/protein B2 of *Alphanodavirus* and *Betanodavirus*. Indeed, Tax and replicases had a codon usage significantly closer to that of the viral genome than the alternative frames, suggesting that they are the ancestral frames. Therefore, the *de novo* frames are those encoding the Rex protein, a post-transcription regulatory factor [32], and the protein B2, a suppressor of RNA silencing [33]. In the four other overlaps, both frames had a comparable codon usage, preventing prediction of genealogy.

The discrepancy between overlapping genes in which the novel frame has a codon usage significantly different from that of the ancestral frame and overlapping genes in which there is no significant difference was investigated by Sabath et al. [34]. They analyzed the evolution of 12 viral genes that arose de novo by overprinting and estimated their relative ages. They found that young de novo genes have a different codon usage from the rest of the genome and that evolve rapidly, under positive or weak purifying selection. In contrast, older de novo genes have a codon usage that is similar to the rest of the genome. They evolve slowly and are under strong purifying selection. Therefore, de novo genes can evolve very rapidly shortly after their origin. As they age, they tend to experience increasingly severe selective constraints, and their codon usage tends to approach that of the ancestral gene from which they originate [34].

To provide a benchmark for systematic studies, Pavesi et al. [35] assembled a high-quality dataset of 80 overlapping genes experimentally proven. They were selected from small or medium-sized eukaryotic viruses with a genome shorter than 30 kb, including single-stranded and double-stranded DNA viruses and single-stranded and double-stranded RNA viruses. The authors found that the overall nucleotide and amino acid composition of overlapping genes is significantly different from that of non-overlapping genes for several composition features. In particular, the proteins they encode show an enrichment in amino acids with high codon degeneracy (the 6-fold degenerate amino acids L, R, and S) and a depletion in amino acids with low codon degeneracy (the 2- and 1-fold degenerate amino acids C, D, E, F, H, K, N, Q, Y, M, and W), a feature that could have been selected because it mitigates the constraints under which the two frames evolve. Using a multivariate statistical method, that is the principal component analysis [36], the study demonstrated that the vast majority of overlapping genes (75 out of 80) follow a similar composition bias, despite their heterogeneity in length and function [35].

A valuable feature of the dataset is that it contains detailed biological information for each pair of overlapping genes (type of experimental evidence for expression, mechanism of translation, function of the two gene products, phenotypic effects upon mutation, and bibliography). By examining this information, Pavesi et al. [35] identified 11 overlaps in which the two encoded proteins take part in the same pathway and interact directly each other. This interaction is critical for viral assembly [37], viral replication [38], relocation of viral genome from nucleus to cytoplasm [39], and viral entry in the host cell [40].

The same study [35] pointed out that the most common mechanisms to express overlapping genes occur at the level of translation. Indeed, more than two thirds of overlapping genes with a known or suspected mechanism of expression (54 out of 71 cases) are expressed by translational processes, such as the use of an alternative start codon [41], ribosomal frameshifting [42], and internal ribosome entry site [43]. The remaining third of overlapping genes is expressed by transcriptional mechanisms, such as the use of sub-genomic RNAs [44] and transcriptional slippage [45].

## 5. Symmetric and Asymmetric Evolution in Viral Overlapping Genes

As first proposed by Miyata and Yasunaga [13], we would expect, in principle, that overlapping genes evolve under strong constraints, because a single nucleotide substitution can simultaneously impair two proteins (e.g., codon position 12 in Figure 1B). An example of “constrained evolution” is that observed in hepatitis B virus (HBV), a small double-stranded DNA virus (3.2 kb) with a high content of overlapping genes. Mizokami et al. [46] found that the mean number of synonymous nucleotide substitutions per site in the five overlapping coding regions of HBV is significantly lower (0.234) than that in non-overlapping regions (0.508).

However, dual-coding genes can also show a less constrained pattern of change, as a consequence of a high rate of non-synonymous substitution in one frame (positive adaptive selection) with concurrent dominance of synonymous substitution in the other (negative purifying selection). In simian immunodeficiency virus, Hughes et al. [47] found that the region of protein Tat under strongest positive selection is encoded by a frame which overlaps, for a length of 150 nt, the frame encoding protein Vpr. Another case is the overlapping gene protein p19/protein p22 (549 nt) of tombusviruses. Allison et al. [48] demonstrated that p19, a suppressor of the host RNA interference mechanism in response to viral infection [49], is under positive selection, whereas p22, a membrane-bound protein essential for cell-to-cell movement of virus [50], is under purifying selection.

These studies suggest that the evolution of overlapping genes can be summarized in accordance to two different models. The first claims that the two proteins encoded by the overlap can evolve under similar selection pressures. In the case of strong selection against amino acid change, both proteins (or protein regions) are highly conserved. For example, comparative analysis of 27 strains of HBV showed that the RNase domain of polymerase and the N-terminal half of protein X have both a percentage of conserved amino acids higher than 90% [46]. In the case of weak selection against amino acid change, both proteins can vary considerably. For example, the same study [46] showed that the spacer domain of polymerase and the pre-S1 region of surface protein show a percentage of conserved amino acids of 30 and 40%, respectively. This model was named “symmetric evolution”, because the number of amino acid substitutions of one protein is expected to be not significantly different from that of the other [51]. It corresponds to the “shared model” described by Fernandes et al. [52].

The other model claims that the two proteins encoded by the overlap can evolve under significantly different selection pressures. Support for this model, which implies positive selection on one frame and negative selection on the other, was provided by a number of studies. In addition to those mentioned previously [47,48], they concern the overlapping gene P/C of Sendai virus [53], the overlapping genes ORF0/ORF1 and ORF3/ORF4 of potato leafroll virus [54], and the overlapping gene VP1/VP2 of human parvovirus B19 [55]. Interestingly, an accordance to this model was also found in the overlapping gene p16INK4a/p19ARF of mammals [56]. This model was named “asymmetric evolution”, because the number of amino acid substitutions of one protein is expected to be significant different from that of the other [51]. It corresponds to the “segregated model” described by Fernandes et al. [52].

As most individual overlapping genes examined in [35] have at least one homolog, I assembled a dataset of 75 pairs of homologous overlaps and analyzed it to determine which of the two evolutionary models is the prevailing one [51]. The study demonstrated that half of overlaps (38 out of 75) evolve in accordance with the asymmetric model. A clear example was the overlapping gene of apple stem grooving virus (ASGV) that encodes a movement protein and a linker-region connecting the RdRp (RNA-dependent RNA polymerase) domain to the coat-protein domain. In detail, the percent amino acid diversity between the linker-region of ASGV and the homolog from citrus tatter leaf virus (39%; 125 differences and 195 identities) resulted to be ten-fold higher than that between the movement protein and the homolog (4%; 13 differences and 307 identities).

The same study [51] pointed out that in all overlapping genes evolving asymmetrically and with known genealogy (23 cases) the most variable protein is that encoded by the de novo frame. Despite the small number of cases, this finding suggests that de novo proteins are the preferred target of selection. As shown in Table 1, most of de novo proteins (14 out of 23) are known to play a role in viral pathogenicity: six act as suppressor of interferon response, four as suppressor of RNA silencing, two as suppressor of interferon response and apoptosis factor, one as apoptosis factor, and one has the ability to selectively degrade the host RNA-polymerase II transcripts. Very interesting is the notion that two *de novo* proteins are known to exert functions that are not virus-specific. They are the apoptin of *Chicken anemia virus*, which induces cell death in a broad range of human tumour cell lines but not in normal cells [57,58], and the protein X of Borna disease virus, which shows protective properties against neurodegeneration in vitro and in vivo [59,60].

Symmetric evolution (similar selection pressures on the two proteins) was found in the remaining 37 overlaps of the dataset [51]. A strong selection against amino acid change was found in the overlapping gene protein 3a/protein 3b of human severe acute respiratory syndrome-related coronavirus (SARS-CoV): the amino acid diversity between protein 3a of human SARS-CoV and the homolog from bat SARS-CoV was rather low (5.3%), as well as that between protein 3b and the homolog (8.8%). A weak selection against amino acid change was found in the overlapping gene of spinach latent virus (SLV) encoding the zinc-finger domain of polymerase and protein 2b: the amino acid diversity between the zinc-finger domain of SLV and the homolog from elm mottle virus was high (47%), as well as that between protein 2b and the homolog (44%).

## 6. Overlapping Genes Show a Peculiar Pattern of Nucleotide and Amino Acid Composition

Overlapping genes represent an unusual pattern of the genetic language [75,76], as two, or exceptionally three, reading frames may lie inside a single nucleotide sequence. The first attempts to detect composition features peculiar to the overlap were carried out using the information theory indices [77]. They are *D*_1_, the divergence from a random nucleotide composition, and *D*_2_, the divergence from a random dinucleotide composition [78,79]. The assumption is that the smallness of *D*_1_, which implies a frequency of each nucleotide around 25%, represents the richness of vocabulary, while the largeness of *D*_2_ represents the clarity of grammatical rules, that is the constraints against a random dinucleotide composition [80]. Thus, information theory predicts that dual-coding genes should have a lower *D*_1_ value and a higher *D*_2_ value when compared to single-coding genes, as hallmarks of a greater information content.

However, comparative analysis of overlapping and non-overlapping genes in the genome of three microviruses (ΦX174, α3 and G4), two avian hepadnaviruses, three strains of HIV-1, two plant luteoviruses, and two plant tymoviruses showed that the pattern predicted by information theory is valid for the first three groups of viruses, but weak for luteoviruses and inconsistent for tymoviruses [17].

In the following years, comparative analyses of overlapping and non-overlapping genes were limited to individual virus species, such as *Infectious bursal disease virus* [81], to virus families such as *Papillomaviridae* [82], or to a small dataset of RNA viruses [21]. Only recently, it was possible to perform a wide-scale analysis using the curated dataset assembled in [35]. It contains, indeed, not only the nucleotide sequence of 80 overlapping genes but also that of the entire complement of non-overlapping genes in the virus genome.

**Figure 5:**
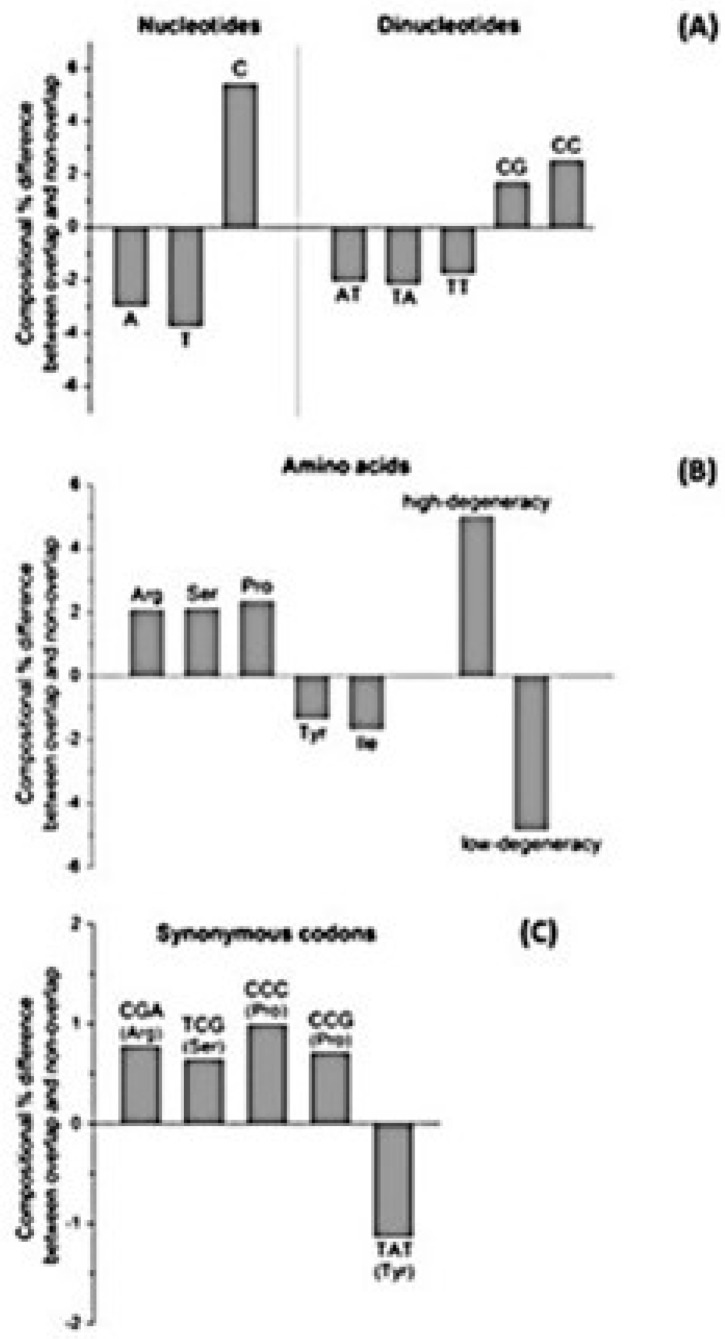
Difference between the pooled sets of overlapping and non-overlapping genes for the 20 most critical composition features. (**A**) Nucleotides and dinucleotides. (**B**) Amino acids and amino acids grouped in accordance to codon degeneracy. (**C**) Synonymous codons. The figure, made by A. Vianelli, was reproduced from [35].

Pavesi et al. [35] found that overlapping genes differ significantly from non-overlapping genes for 20 composition features (Figure 5). Some of them are clearly linked. For example, the enrichment in C of overlapping genes is linked to that in dinucleotide CC, codons CCC and CCG, and proline. The depletion in A and T of overlapping genes is linked to that in amino acids with a low codon degeneracy, because they are encoded by codons rich in A and T. Depletion in T, A, and TA of overlapping genes reduces the probability of occurrence of stop codons (TGA, TAG and TAA) and thereby increases that of occurrence of long overlapping frames.

The dataset in [35] was also a valuable start point to assemble a much larger one [83]. For each overlapping gene, it included all the homologs gathered from the NCBI Viral Genome Database [84]. The size of the sample increased from 80 to 319 overlaps, coming from 244 virus species (the number of virus species is lower than that of overlaps because some viruses contain more than one overlap). Consider for example the overlapping gene replicase/movement protein of tymoviruses. The dataset in [35] contains only the overlap of turnip yellow mosaic virus (TYMV), the dataset in [51] contains the overlap of TYMV and the homolog of watercress white vein virus (nucleotide diversity of 28%), while the dataset in [83] contains as many as 20 homologous overlaps, covering a nucleotide diversity from 28 to 50%.

**Figure 6:**
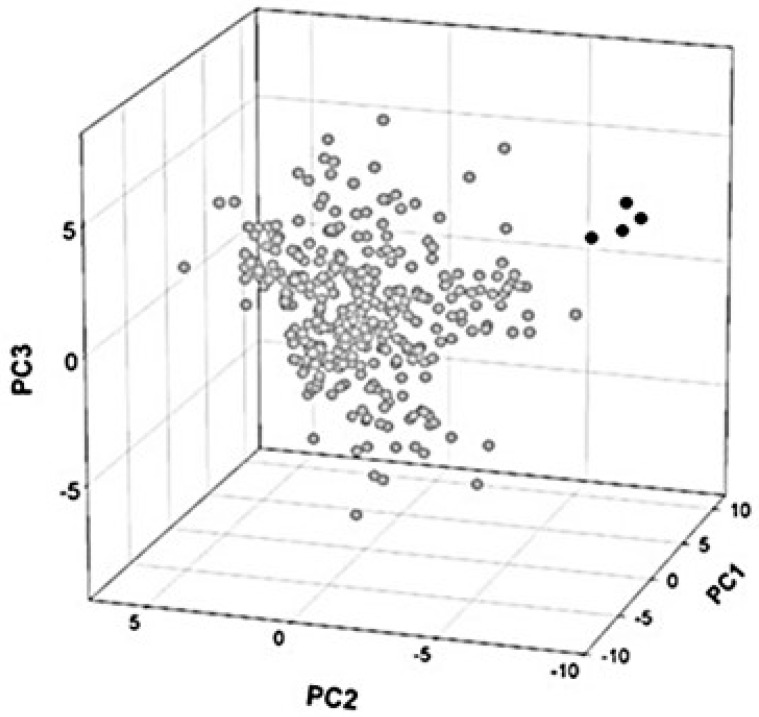
Principal component analysis (PCA) of a sample set of 319 overlapping genes. The three-dimensional map was obtained using the first (PC1), second (PC2), and third (PC3) principal component. Black circles indicate the 4 homologs of the overlapping gene polymerase/protein X of Hepatitis B virus. They were classified as outlier because of a highly atypical sequence composition. Figure reproduced from [83] with the permission of Elsevier.

By comparative analysis of overlapping and non-overlapping genes (319 overlaps and 244 non-overlaps), I detected a total of 37 significantly different composition features [83]. Principal component analysis, aimed to evaluate whether the observed differences were homogeneously distributed in individual overlapping genes, revealed the presence of only four outliers (Figure 6). This finding confirmed that overlapping genes follow a common pattern of composition bias, despite their different length and function.

With the aim to distinguish overlapping from non-overlapping genes with the best accuracy, I compared the sample set of 319 overlaps to the control-set of 244 non-overlaps using multivariate statistics [83]. The methods were the Fisher’s linear discriminant analysis (LDA) [85,86] and the partial least squares-discriminant analysis (PLS-DA) [87,88].

**Figure 7:**
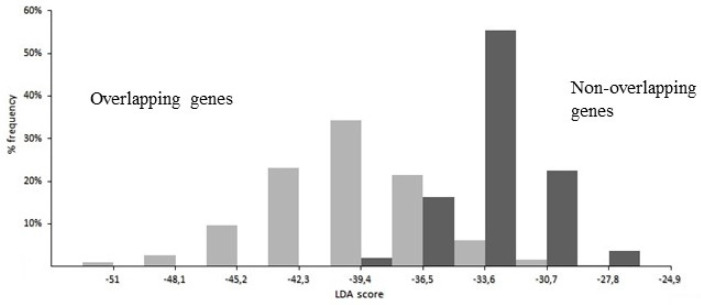
Histogram of the distribution of LDA score in overlapping genes (grey columns) and in non-overlapping genes (black columns). With a discriminant score of −35.31, a high percentage (96.5%) of overlapping genes were correctly classified as overlap (score below −35.31) and a high percentage (97.1%) of non-overlapping genes were correctly classified as non-overlap (score above −35.31). Figure was reproduced from [83] with the permission of Elsevier.

The best performance of LDA was given by a linear function of 21 coefficients, corresponding to 21 significantly different composition features between overlap and non-overlap (two from nucleotides, four from dinucleotides, eight from amino acids, and seven from synonymous codons). As shown in Figure 7, the strong discriminant power of the function is highlighted by the different distribution of the LDA score in overlapping genes (grey columns) compared to that in non-overlapping genes (black columns).

**Figure 8:**
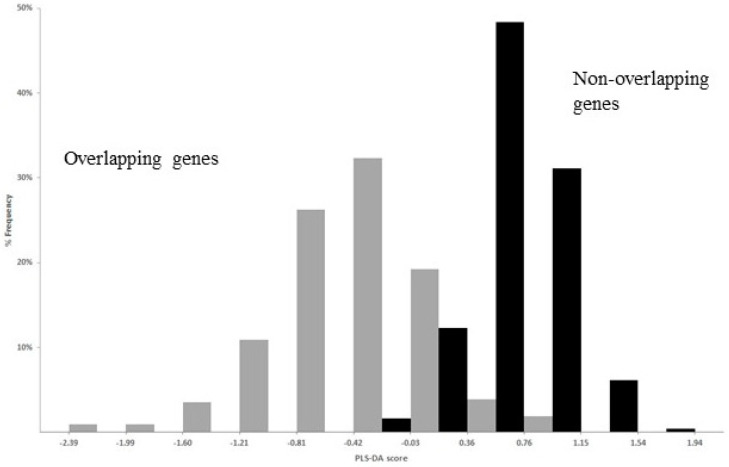
Histogram of the distribution of PLS-DA score in overlapping genes (grey columns) and in non-overlapping genes (black columns). With a discriminant score of 0, a high percentage of overlapping genes (94.9%) were correctly classified as overlap (score below 0) and a high percentage (98.4) of non-overlapping genes were correctly classified as non-overlap (score above 0). Figure reproduced from [83] with the permission of Elsevier.

The best performance of PLS-DA was given by a linear regression function of 23 regression coefficients, corresponding to 23 significantly different composition features between overlap and non-overlap (one from nucleotides, six from dinucleotides, seven from amino acids, and nine from synonymous codons). The strong discriminant power of the function is evident in Figure 8, which shows the distribution of the PLS-DA score in overlapping (grey columns) and non-overlapping genes (black columns).

## 7. Birth of Overlapping Genes in Viruses: Gene Compression or Gene Novelty?

The abundance of overlapping genes in viruses [89] was explained by two, not mutually exclusive, theories. The gene-compression theory states that the gene overlap is a valuable strategy to maximize the coding ability of small genomes [13,17,90,91,92], as a consequence of biophysical constraints on the size of the capsid structure [93] or of a high mutation rate such that occurring in RNA viruses [94]. As most mutations are deleterious, the high mutation rate will limit the genome size, and thus new genes must come from overprinting [95]. The gene-novelty theory claims that the birth of novel proteins by overprinting is driven by selection pressures providing the virus with a fitness advantage that lead to their fixation [9,21,96].

Using as benchmark the dataset of overlapping genes assembled in [83], I could determine which of the two theories is the most plausible one. Using the phylogenetic and codon-usage criteria, I first predicted the genealogy of 46 overlapping genes. By extending the inferred genealogy to the homologs, I then obtained a dataset of 194 overlapping genes with a known ancestral and de novo frame: 126 overlaps with a +1 de novo frame and 68 overlaps with a +2 de novo frame. Analysis of amino acid and synonymous codon composition revealed that the +1 and +2 de novo frames differ significantly from the respective ancestral frames for 25 and 23 composition features, respectively [83].

**Figure 9:**
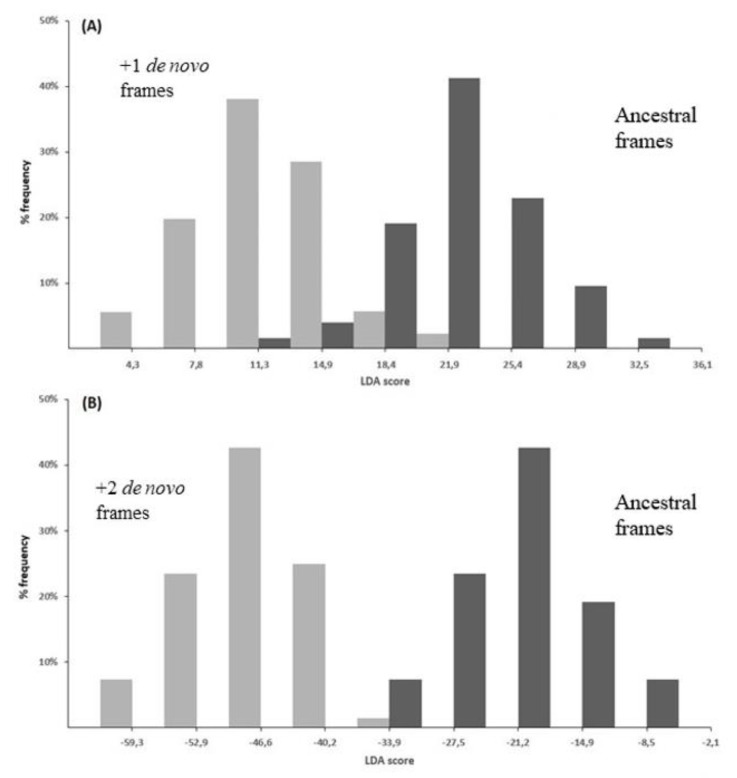
(**A**) Histogram of the distribution of the LDA score in 126 ancestral frames (black columns) and in the respective +1 de novo frames (grey columns). With a discriminant score of 17.20, a high percentage (96.8%) of ancestral frames were correctly classified as ancestral (score above 17.20) and a high percentage (97.6%) of +1 de novo frames were correctly classified as de novo (score below 17.20). (**B**) Histogram of the distribution of the LDA score in 68 ancestral frames (black columns) and in the respective +2 de novo frames (grey columns). With a discriminant score of −34.98, all ancestral frames and all +2 de novo frames were correctly classified as ancestral and de novo, respectively. Figure reproduced from [83] with the permission of Elsevier.

On the basis of these differences in composition, the linear discriminant analysis clearly separated the ancestral frames from the +1 de novo frames (Figure 9A), as well as the ancestral frames from the +2 de novo frames (Figure 9B). When compared to the respective ancestral proteins, the +1 de novo proteins were found enriched in hydrophobic residues and depleted in acidic residues, while the +2 de novo proteins were found enriched in basic residues and cysteine and depleted in hydrophobic residues [83]. Although one theory does not entirely exclude the other, the different amino acid composition of de novo proteins vs. the ancestral ones should better support gene-novelty than gene-compression.

In the same study [83], I examined the 244 virus species in the dataset to determine whether there is a negative relationship between the length of their genomes and that of their overlapping genes, a feature in accordance to the gene-compression theory. Using the Spearman rank correlation coefficient, I found a significant negative correlation of −0.31, too weak however for supporting the gene-compression theory. A similar study demonstrated that gene overlap is not a significant factor in the compression of viral genomes [96].

## 8. Modular Evolution in Overlapping Genes: The Case of Hepatitis B Virus

The theory of modular evolution for viruses predicts that various coding sequences are used as functional modules during recombination events [97]. This is thought to speed up virus evolution by utilizing various combinations of functional modules to gain novel genes [98,99]. However, viruses can also evolve through a mechanism in which the gain of novel modules depends on overprinting. Two studies showed that modular evolution played a critical role in the genesis of the overlapping gene polymerase/surface protein of hepadnaviruses [100,101]. 

**Figure 10:**
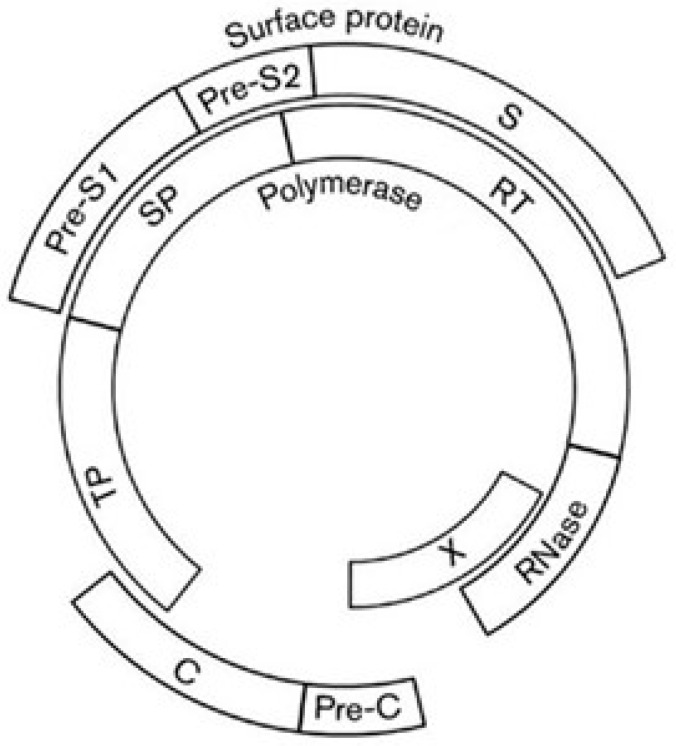
Map of the genome of HBV with overlapping and non-overlapping coding regions. Pre-S1, Pre-S2, and S are the domains of surface protein. TP, SP, RT, and RNase are the domains of polymerase. TP, terminal protein domain; SP, spacer domain; RT, reverse transcriptase domain; RNase, ribonuclease domain; C, capsid. Figure reproduced from [100] with the permission of the Microbiology Society.

Hepatitis B virus (HBV), a member of the familiy *Hepadnaviridae*, is a DNA reverse-transcribing virus with a circular genome of 3.2 kb. About 50% of the genome contains overlapping coding regions, due to the large overlap between the gene for polymerase (P) and the genes for capsid (C), X, and surface (S) proteins (Figure 10).

Several studies were carried out to investigate the role of gene overlap in the evolution of HBV [46,102,103,104,105]. The genetic diversity of the overlapping proteins P and S was also related to virus survival in response to antiviral drugs [106], to virus escape from neutralizing antibodies [107], and to the clinical significance of mutations induced by selection [108].

Using the phylogenetic method, the genealogy of the overlap between the RNase domain of polymerase and the N-terminal half of protein X was clearly elucidated. The method predicts that protein X arose de novo, because of its presence in *Orthohepadnavirus* but not in the sister genus *Avihepadnavirus* [21,109]. In contrast, the genealogy of the overlap between the surface protein and the spacer (SP) and reverse-transcriptase (RT) domains of polymerase was difficult to predict. In this case, the phylogenetic criterion was not applicable because the homologs of both frames show an identical phylogenetic distribution, making possible only the codon-usage approach.

**Figure 11:**
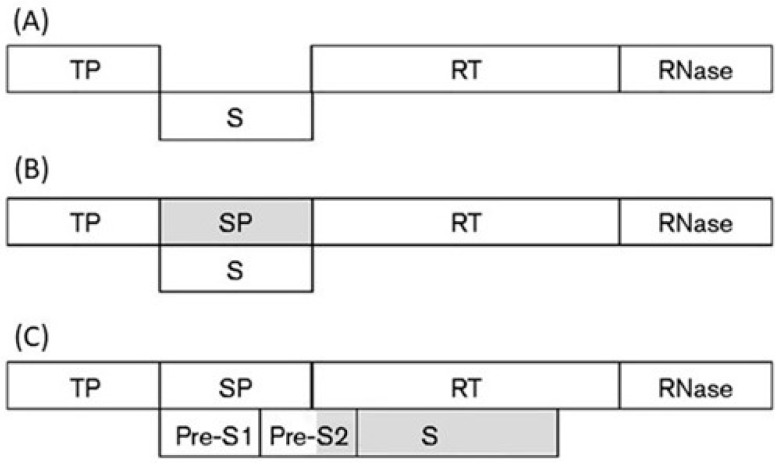
Modular evolution in the genesis of the overlapping gene polymerase/surface protein of hepadnaviruses. (**A**) Putative primordial genome of HBV. (**B**) Birth of a novel frame encoding the SP domain of polymerase (shaded box). (**C**) Birth of a novel frame encoding the C-terminal region of the Pre-S2 domain and the S domain of surface protein (shaded box). Figure reproduced from [100] with the permission of the Microbiology Society.

By a sliding-window analysis of the codon usage along the entire overlapping coding region (1200 nt), I found that the overlap P/S can be subdivided into two regions, each with its own pattern of codon usage [100]. By predicting the ancestral and the de novo frame in each region, I hypothesized a primordial genome with a short gene S placed between the gene encoding the terminal protein (TP) and the gene encoding the RT and RNase domains of polymerase (Figure 11A). A first increase in coding density was due to the birth, within gene S, of a de novo frame encoding the spacer (SP) domain (Figure 11B). Acting as linker, it led to creation of a multi-domain polymerase (TP, SP, RT, and RNase domains).

A further increase in coding density was due to a long overlapping extension of gene S. In addition to a full-length Pre-S2 domain, it led to a de novo creation of the S domain of surface protein (Figure 11C). As a result, this overlapping extension generated a surface gene consisting of three in-phase ORFs, whose co-translation yields the large surface protein. Taken together, these evolutionary inferences suggest that the overlapping gene polymerase/surface protein attained its present complexity through modular evolution [100].

The hypothesis that the Pre-S/S ORF is an innovation unique to the hepadnaviral lineage was confirmed by Lauber et al. [101]. In addition, they dated the de novo emergence of Pre-S/S about 400 million years ago. This date corresponds to the inferred separation time between hepadnaviruses (enveloped viruses with a surface-protein gene) and nackednaviruses (non-enveloped fish viruses lacking a surface-protein gene). Both studies [100,101] pointed out that overprinting is a source not only of de novo accessory proteins with regulatory function [20,21,22,23,24,25,26,27,28], but also of de novo essential structural proteins, such as the large surface protein of hepadnaviruses.

## 9. Estimation of Selection Intensities in Overlapping Genes by *ad hoc* Methods

The strength of selection pressure in protein-coding genes is usually inferred by comparing the number of non-synonymous nucleotide substitutions per site (d_n_) with that of synonymous nucleotide substitutions per site (d_s_), with d_n_/d_s_ > 1 indicative of positive selection and d_n_/d_s_ < 1 of negative selection [110,111]. Extending this standard approach to overlapping genes is inappropriate, because a nucleotide substitution that is synonymous in one frame is highly likely to be non-synonymous in the alternative frame. It follows that the constraints against synonymous substitutions in a frame significantly lowers its d_s_ value, causing an artifactual increase of d_n_/d_s_ and a wrong inference of positive selection if d_n_/d_s_ > 1.

To overcome this problem, several researchers have developed methods for correctly estimating the strength of selection intensities in overlapping genes. The maximum-likelihood model by Hein and Støvlbæk [112] was an extension of the notion of degeneracy class of a site [111] to that of a combination of two degeneracy classes (one for each frame to which a site belongs). De Groot et al. integrated this model into a statistical alignment framework and estimated selection in the overlapping genes of HBV and HIV-2 [113]. McCauley et al. developed a Hidden Markov Model (HMM) capable of accounting for varying levels of selection along the viral genome, including those acting on overlapping ORFs [114]. When applied to a multiple alignment of HIV-2 sequences, HMM was able to make truly statistically significant statements about the nature of selection on dual-coding regions. The Markov-chain Monte Carlo model by Pedersen and Jensen [115] incorporated the constraints imposed by both of the overlapping genetic codes in an exact manner. This model, indeed, included parameters representing the degrees of selection constraints operating in the different frames.

Sabath et al. proposed a non-stationary method, similar to that of Pedersen and Jensen but with the advantage to avoid the need for computationally-expensive procedure [116]. The method was tested on the overlapping genes PB1-F2 and NS1 of influenza A virus, because they were previously reported to exhibit values of d_n_/d_s_ remarkably higher than 1 (9.4 for PB1-F2 and 1.9 for NS1) and thus indicative of strong positive selection [117,118]. The method demonstrated that PB1-F2 and NS1 appear to be under weak negative selection, because of a d_n_/d_s_ value of 0.50 and 0.70 respectively. Therefore, the previous estimates of selection on PB1-F2 and NS1 were wrong, because they were calculated ignoring the interdependence with the respective overlapping frames PB1 and NS2. A limitation of the Sabath’s method is that it restricts the analysis to homologous overlapping genes in which the two encoded proteins have both an amino acid diversity smaller than 50% or greater than 5%.

The method developed by Wei and Zhang [119] was an extension of the standard method for protein-coding genes originally proposed by Nei and Gojobori [111]. The method first classifies each site in the reference overlapping gene into four categories (NN, NS, SN, and SS, where N stands for non-synonymous and S for synonymous), depending on the impacts of potential mutations on the two overlapping ORFs (ORF1 and ORF2). The method then classifies all nucleotide differences between the reference overlapping gene and its homolog into four categories (NN, NS, SN, and SS) and counts their numbers (M_NN_, M_NS_, M_SN_, and M_SS_, respectively). Finally, the method estimates the strength of natural selection acting on ORF1 by ω_1_ = d_NN_/d_SN_ and that acting on ORF2 by ω_2_ = d_NN_/d_NS_.

## 10. Computational Methods to Predict Overlapping Genes in Viruses

To identify overlapping genes by sequence analysis, several groups have developed methods that detect the atypical pattern of nucleotide substitution induced by the overlap. Firth and Brown developed a method called Maximum-Likelihood Overlapping Gene Detector (MLOGD), which was designed to detect the mutation signature of overlapping coding sequences in pairwise alignments of two sequences, under a double-coding model [120]. The same authors presented an improved version of MLOGD, whose ability to estimate the magnitude of constraints on the gene overlap yielded a sensitivity of 90% in the detection of known overlapping genes [121].

A further improvement was provided by the computational tool Synplot2 [122]. It analyzed alignments of protein-coding virus sequences to identify regions where there is a statistically significant reduction in the degree of variability at synonymous sites, a characteristic signature of overlapping functional elements such an overlapping gene or a conserved RNA structure. The same approach was followed by Sealfon et al., who developed a phylogenetic codon-model based method (FRESCo, that is Finding Regions of Excess Synonymous Constraints) for detecting virus regions with a significantly reduced synonymous variability [123]. When applied to a multiple alignment of over 2000 whole-genome sequences of HBV, FRESCo detected strong synonymous constraint elements within known regions of overlapping function (overlapping ORFs or regulatory elements).

By modifying the method in [119], Nelson et al. [124] developed a computational tool named OLGenie, where OLG means OverLapping Gene. It estimated signs of strong purifying (negative) selection in aligned sequences as hallmark of functional overlapping genes. Assessment with simulations and controls from viral genomes (58 OLGs and 176 non-OLGs) demonstrated low false-positive rates and good ability in differentiating true OLGs from non-OLGs.

Although powerful, these computational methods are necessarily constrained by the requirement for multiple sequences of sufficient diversity to reliably detect overlapping genes. Therefore, these methods are not applicable in the case of a single nucleotide sequence or sequences with a low nucleotide diversity. To overcome this drawback, Schlub et al. developed a statistical method that relies on only a single gene, or genome, nucleotide sequence [125]. The method detects candidate overlapping genes in viruses by selecting overlapping ORFs that are significantly longer than expected by chance. It consists of a codon-permutation test and a synonymous-mutation test. The limit of the method is that the sensitivity was high (90% for codon-permutation test and 95% for synonymous-mutation test) for overlapping genes longer than 300 nt, but rather low for those longer than 100 nt (65% for codon-permutation test and 71% for synonymous-mutation test).

**Figure 12:**
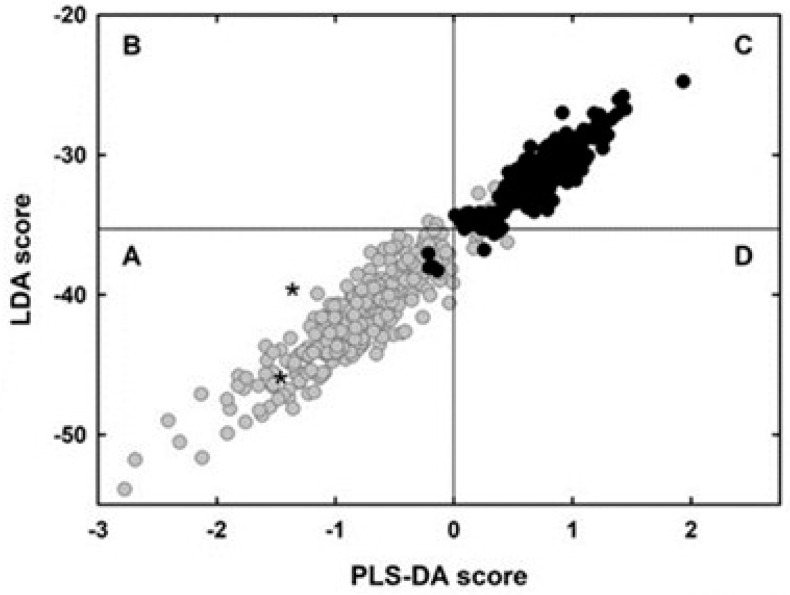
Map of overlapping genes (grey circles) and non-overlapping genes (black circles), in which the PLS-DA score is plotted against the respective LDA score. Grey circles in part (**A**) indicate overlaps correctly classified by both methods (94.2% of the total). Black circles in part C indicate non-overlaps correctly classified by both methods (97.1% of the total). Gray circles in part (**B**–**D**) indicate overlaps misclassified by one or both methods (5.8% of the total). Black circles in part (**A**) and (**D**) indicate non-overlaps misclassified by one or both methods (2.9% of the total). Asterisks in part (**A**) indicate two new potential overlapping genes detected in the genome of SARS-CoV-2 (isolate Wuhan-Hu-1). Figure reproduced from [83] with the permission of Elsevier.

Another prediction method that relies on single nucleotide sequences was the combined use of linear discriminant analysis (LDA) and partial least squares-discriminant analysis (PLS-DA) [83]. Taken individually, LDA correctly classified 96.5% of overlapping genes and 97.1% of non-overlapping genes (Figure 7) and PLS-DA 94.9% of overlapping genes and 98.4% of non-overlapping genes (Figure 8). The performance of the combined use of LDA and PLS-DA is summarized in Figure 12. Grey circles in part A indicate the overlaps correctly classified by both methods (94.2% of the total). Black circles in part C indicate the non-overlaps correctly classified by both methods (97.1% of the total). Application of the method to the genome sequence of SARS-CoV-2 (isolate Wuhan-Hu-1), the etiological agent of current pandemic [126], led to detection of two new potential overlapping ORFs (asterisks in part A of the figure).

Another method analyzing single, or closely related, genome sequences was GOFIX [127]. It detects overlapping ORFs on the basis of a significant enrichment in the X motif (a set of 20 codons over-represented in viral genes).

## 11. Brief Note on the Presence of Overlapping Genes in Prokaryotes and Eukaryotes

Although the present review is focused on viral overlapping genes, it is important to note that experimental and computational reports suggest that the birth of new genes by overprinting is not confined to viruses. It is a much wider phenomenon than previously thought, both in prokaryotic [128,129] and eukaryotic genomes [130,131,132,133,134,135]. Thus, the expression of two proteins from the same mRNA has changed the traditional view that a mature eukaryotic mRNA is a mono-cistronic molecule with a single translated ORF [136,137]. Interestingly, it has also been found that some human cancer-specific antigens, silent in normal tissues, are translated from alternative open reading frames (AltORFs) [138,139,140,141,142]. These neoantigens are promising targets for the development of anti-tumour immunotherapies with a potentially broader coverage of patients [143].

## 12. Brief Note on the Presence of Anti-Sense Overlapping Genes in Viruses

Overlapping genes can be classified broadly into two types: (1) same-strand overlapping genes, which are transcribed from the same strand of DNA (also known as sense-overlap); (2) different-strand overlapping genes, which are transcribed from two opposite strands of DNA (also known as anti-sense overlap).

As the great majority of known overlapping genes are of same-strand type, they were the primary focus this review. However, I would briefly report two cases of anti-sense overlap experimentally validated. The first was found in the pX region of Human T-lymphotropic virus 1 (HTLV-1). The sense strand encodes p30, a protein playing a role in viral replication, host immunity, and cellular proliferation [144]. The anti-sense strand encodes HBZ, a transcription factor playing a critical role in HTLV-1 associated diseases [145,146]. Because the pX region of HTLV-1 also contains the sense-overlap Tax protein/Rex protein, it constitutes a hotspot of gene origination, or gene “nursery” [147]. Its complex pattern of origin and evolution is accurately presented in [31].

The other anti-sense coding sequence, termed ASP and overlapping the gene Env, was predicted in HIV-1 by Cassan et al. [148]. Using computer simulations, they showed that conservation of ASP in HIV-1 (specifically in the group M) could not be due to chance but to selection pressure conserving the start codon and avoiding stop codons. Affram et al. demonstrated the presence of the ASP protein on the surfaces of both infected cells and viral particles, yielding evidence that this accessory protein is a new structural component of HIV-1 [149].

## 13. Concluding Remarks and Future Directions

Over four decades after the discovery of overlapping genes [11,12], we have an accurate knowledge of their origin and evolution. This review highlights that *de novo* protein creation by overprinting is a significant factor in viral evolution, in particular in the evolution of pathogenicity. At the same time, it is a valuable start point for future studies.

For example, factors affecting the birth of overlapping genes can be further investigated by a sequence-composition analysis of “pre-overlapping coding regions”, that is the genome regions homologous to a gene overlap but lacking it. This analysis could assess if the composition bias is a contributing factor (i.e., a cause) to the existence of overlapping genes or a consequence of selection acting on overlapping genes after they are born.

The accuracy of multivariate statistics (LDA and PLS-DA) in determining whether a candidate overlapping ORF is coding or non-coding can be improved by comparing the sample set of overlapping genes to a control set of spurious overlapping genes, rather than of non-overlapping genes (a spurious overlapping gene is a protein-coding region that overlaps purely by chance an ORF not interrupted by stop codon).

Having found that a small set of mammalian overlapping genes follows a composition bias similar to viral one [35], a few prediction methods could be used to detect overlapping genes in eukaryotic genome sequences. They probably contain numerous undetected overlapping genes, as suggested by increasing experimental evidence [136]. Because stop codons (TGA, TAG, and TAA) are GC-poor, overlapping genes are expected to occur less frequently by chance in eukaryotic GC-rich sequences [150]. Theoretical studies focused on constraints (and their combinatorics) acting on the amino acid composition of paired overlapping proteins may form the basis for a quick and simple method to detect overlapping regions within proteins [151,152,153].

The computational methods reported in Section 10 are also a valuable tool to detect new potential overlapping genes in the NCBI Viral Genome Database (e.g., in large DNA viruses), to include in database proven overlaps overlooked during genome annotation, or to exclude hypothetical overlaps that may be artefacts of genome annotation.

The wide collection of proven overlapping genes and their homologs [35,83] can be used by others as reference datasets for further studies. They could expand our knowledge about their relative age, thus increasing the number of known cases of oldest and youngest *de novo* overlapping genes. They could test the occurrence of symmetric/asymmetric evolution in different regions of the same overlapping gene, as done for example in the overlap Tat protein/Rev protein of HIV-1 [52]. The relationship between gene overlap and evolutionary rate, investigated in RNA viruses [154], could be extended to DNA viruses.

A web server, called Coevolution in OVerlapped sequences by Tree analysis (COVTree), has been developed recently by Teppa et al. [155]. COVTree analyzes the effect of mutations in one protein over the other and detects coevolution signals in “mirrored” positions. It could be applied to the large dataset of homologous overlapping genes assembled in [83].

As viral protein synthesis is completely dependent upon the translational machinery of the eukaryotic host cell, studying overlapping genes has greatly improved our knowledge of gene expression. Indeed, non-canonical translational strategies such as leaky scanning, ribosomal frameshifting and alternative initiation are essential for expression of overlapping genes [41,42,43,44,45,156]. Therefore, detection of overlapping genes in eukaryotes may further improve our knowledge of gene expression by translational recoding [157].

Finally, the finding that a few de novo proteins have previously unknown 3D structural folds [158,159] and mechanisms of action [160] suggests that overlapping genes provide powerful model systems to test ideas about protein folding and evolution.

## Figures and Tables

**Table 1:** List of 14 overlapping genes evolving asymmetrically and with a known function of the *de novo* protein.

Virus Species and Genome Ac. Number	Overlapping Gene (Protein Products)	Predicted *De Novo* Protein (Prediction Criterion)	Most Variable Protein (Length of Overlap)	Function [Bibliographic Reference]
Theiler’s murine encephalomyelitis virus (NC_001366)	Polyprotein region encoding the leader and VP4 capsid proteins/protein L*	Protein L* (phylogeny and codon usage)	Protein L* (156 aa)	Suppressor of interferon response [61]
Hepatitis C virus (NC_004102)	Polyprotein region encoding the core protein/protein F	Protein F (codon usage)	Protein F (151 aa)	Suppressor of interferon response [62]
Puumala virus (NC_005224)	Nucleocapsid protein/non-structural protein NSs	Non-structural protein NSs (codon usage)	Non-structural protein NSs (90 aa)	Suppressor of interferon response [63]
Infectious pancreatic necrosis virus (NC_001915)	Protein VP5/polyprotein region encoding the N-half of capsid protein VP2	Protein VP5 (phylogeny and codon usage)	Protein VP5 (131 aa)	Suppressor of interferon response [64]
Borna disease virus (NC_001607)	Protein X/phosphoprotein (P)	Protein X (codon usage)	Protein X (71 aa)	Suppressor of interferon response [65]
Infectious salmon anemia virus (NC_006497)	Protein p6/protein p7	Protein p6 (codon usage)	Protein p6 (183 aa)	Suppressor of interferon response [66]
Apple chlorotic leaf spot virus (NC_001409)	Protein p50/capsid protein	Protein p50 (phylogeny)	Protein p50 (105 aa)	Suppressor of RNA silencing [23]
Tomato bushy stunt virus (NC_001554)	Protein p19/protein p22	Protein p19 (phylogeny)	Protein p19 (172 aa)	Suppressor of RNA silencing [67]
Turnip yellow mosaic virus (NC_004063)	Protein p69/replicase (methyltransferase domain and downstream region)	Protein p69 (phylogeny and codon usage)	Protein p69 (626 aa)	Suppressor of RNA silencing [68]
East African cassava mosaic virus (NC_004674)	Protein AC1/protein AC4	Protein AC4 (phylogeny)	Protein AC4 (77 aa)	Suppressor of RNA silencing [69]
Murine norovirus (NC_008311)	Capsid protein VP1/virulence factor VF1	Virulence factor VF1 (phylogeny and codon usage)	Virulence factor VF1 (213 aa)	Suppressor of interferon response and apoptosis factor [70]
Influenza A virus (NC_002021)	Subunit PB1 of RdRp/protein PB1-F2	Protein PB1-F2 (phylogeny and codon usage)	Protein PB1-F2 (87 aa)	Suppressor of interferon response and apoptosis factor [71,72]
Chicken anemia virus (NC_001427)	Capsid protein VP4/apoptin	Apoptin (phylogeny)	Apoptin (119 aa)	Apoptosis factor [73]
Influenza A virus (NC_002022)	Subunit PA of RdRp/protein PA-X	Protein PA-X (codon usage)	Protein PA-X (61 aa)	Degradation of the host RNA-polymerase II transcripts [74]

## Data Availability

The data presented in this study are available on request from the corresponding author.
